# Effect of Temperature-Dependent Low Oxygen Partial Pressure Annealing on SiC MOS

**DOI:** 10.3390/nano14020192

**Published:** 2024-01-15

**Authors:** Qian Zhang, Nannan You, Jiayi Wang, Yang Xu, Kuo Zhang, Shengkai Wang

**Affiliations:** 1 High-Frequency High-Voltage Device and Integrated Circuits R&D Center, Institute of Microelectronics of the Chinese Academy of Sciences, Beijing 100029, China; zhangqian@ime.ac.cn (Q.Z.); zhangkuo@ime.ac.cn (K.Z.); 2 University of Chinese Academy of Sciences, Beijing 100049, China

**Keywords:** silicon carbide, interface state density, low oxygen partial pressure, temperatures, ToF-SIMS

## Abstract

Oxygen post annealing is a promising method for improving the quality of the SiC metal oxide semiconductor (MOS) interface without the introduction of foreign atoms. In addition, a low oxygen partial pressure annealing atmosphere would prevent the additional oxidation of SiC, inhibiting the generation of new defects. This work focuses on the effect and mechanism of low oxygen partial pressure annealing at different temperatures (900–1250 °C) in the SiO_2_/SiC stack. N_2_ was used as a protective gas to achieve the low oxygen partial pressure annealing atmosphere. X-ray photoelectron spectroscopy (XPS) characterization was carried out to confirm that there are no N atoms at or near the interface. Based on the reduction in interface trap density (D_it_) and border trap density (N_bt_), low oxygen partial pressure annealing is proven to be an effective method in improving the interface quality. Vacuum annealing results and time of flight secondary ion mass spectrometry (ToF-SIMS) results reveal that the oxygen vacancy (V[O]) filling near the interface is the dominant annealing mechanism. The V[O] near the interface is filled more by O_2_ in the annealing atmosphere with the increase in temperature.

## 1. Introduction

Silicon carbide (SiC) has been attracting wide attention because of its excellent physical properties, such as wide bandgap, high thermal conductivity, and high breakdown field [1,2,3]. On this basis, SiC metal oxide semiconductor field effect transistors (MOSFETs) are also promising power devices with low loss and fast switching [3,4,5]. However, the interface state density (D_it_) near the SiC conduction band edge (E_C_) of SiC MOSFETs is more than an order of magnitude higher than that of Si MOSFETs. Consequently, the electrical characteristics of the SiC MOSFETs are limited by the existence of the high D_it_, which could be attributed to the generation of C defects in the SiC oxidation process [6,7,8].

Many studies have shown that the D_it_ of the SiO_2_/SiC stack can be reduced by optimizing the oxidation and post oxidation annealing (POA) processes. For example, the oxidation of the deposited Si on the SiC layer instead of direct oxidation of SiC can minimize the carbon-related defects in the oxide layer [9]. In addition, high-pressure microwave plasma oxidation is designed to promote a more complete interface oxidation reaction using atomic oxygen species [10]. Meanwhile, POA in different atmospheres for SiC MOS capacitors, such as POCl_3_ [11], N_2_ [12], NO [13], N_2_O [14], etc., are proposed. The key to these annealing methods is to introduce foreign passivating atoms into the SiO_2_/SiC stacks to passivate the interface. For example, N atoms can passivate the C-related defects at the interface mainly by replacing some C atoms to form stable Si-N bonds [15,16]. However, the passivation atoms may induce fast states or hole traps [17], which can lead to reliability problems. Therefore, oxygen is an alternative atmosphere in the POA process. We have reported that the O_2_ POA can improve the quality of the SiC MOS without introducing any foreign atoms. The deconvolution analysis of X-ray photoelectron spectroscopy (XPS) of Si 2p spectra at different distances from the interface reveals that O atoms fill the oxygen vacancy at and near the interface [18]. In order to avoid further oxidation of SiC induced by excess oxygen during the annealing process, a relatively low oxygen partial pressure should be selected [19]. Considering the oxygen vacancy filling process, the effect of annealing at low oxygen partial pressure is highly dependent on the temperature. T. Kobayashi et al. reported that low oxygen partial pressure annealing at high temperature (≥1300 °C) can reduce the D_it_ value and prevent oxide layer degradation [19]. However, few studies have pointed out the annealing effect of low oxygen partial pressure at temperatures below 1300 °C.

In this paper, we focus on the temperature range from 900 to 1250 °C to study the effect of temperature on low oxygen partial pressure annealing. The N_2_ is utilized as a protective gas to achieve an annealing atmosphere with a low oxygen partial pressure. Electrical characteristics were measured to analyze the annealing effect, and the mechanism of the corresponding temperature range was revealed by the time of flight secondary ion mass spectrometry (ToF-SIMS) characterization.

## 2. Materials and Methods

**Figure 1:**
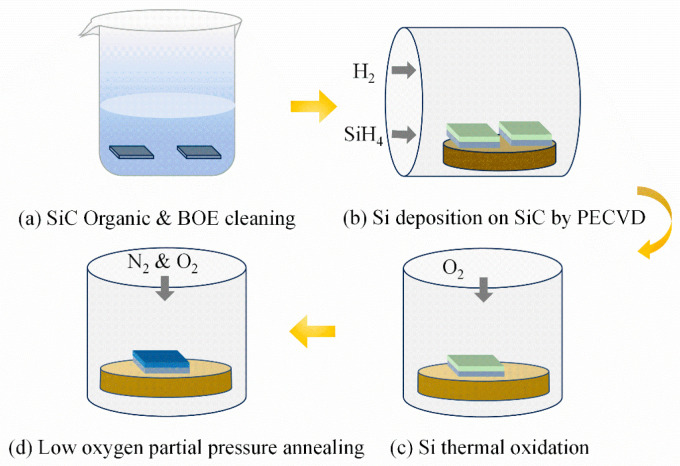
Schematic diagram of SiO_2_/SiC structure formation and annealing process.

The procedures of the oxide layer formation and annealing process are shown in Figure 1. The N-type (8.0 × 10^15^ cm^−3^) 4H-SiC (0001) epitaxial layer was used in this work. After successive organic and buffered oxide etch (BOE, HF: NH_4_F = 1:7) cleaning, Si was deposited on the 4H-SiC wafer by introducing silane (SiH_4_) and H_2_ under 90 Pa at 200 °C for 8 min. The samples were oxidized in O_2,_ resulting in an oxide layer with a thickness of 35 nm, which was calculated by C-V curves. Then, the oxidized samples were annealed in the mixture gas of N_2_ and O_2_ at 900, 1000, 1100, 1200, and 1250 °C for 10 min, respectively, in which the oxygen partial pressure was 0.01 Pa. To investigate the annealing mechanism at low oxygen partial pressure, the control samples were annealed in a vacuum (3 × 10^−3^ Pa). Finally, the Al electrode was deposited to form the SiC MOS capacitors.

The capacitance–voltage (C-V) curves were characterized by Keysight E4990 LCR. The high–low method was used to calculate the interface state density (D_it_) [20], which represents the number of defects at the interface. The change in the fixed charge density in the oxide layer during annealing was observed with the ideal flat band voltage (V_FB_) [21,22] as the reference. XPS measurement was performed to analyze the composition of oxide layer elements. The ESCALAB 250Xi system carrying a monochromatic K_α_ line from an Al anode of 197 W was used to collect spectra. In addition, ToF-SIMS was performed to characterize the element distribution of the oxide layer in a more accurate way. The ToF-SIMS 5–100 instrument equipped with a Cs^+^ 1 keV ion beam for depth profiling was used to characterize the element distribution of oxide layers.

**Figure 2:**
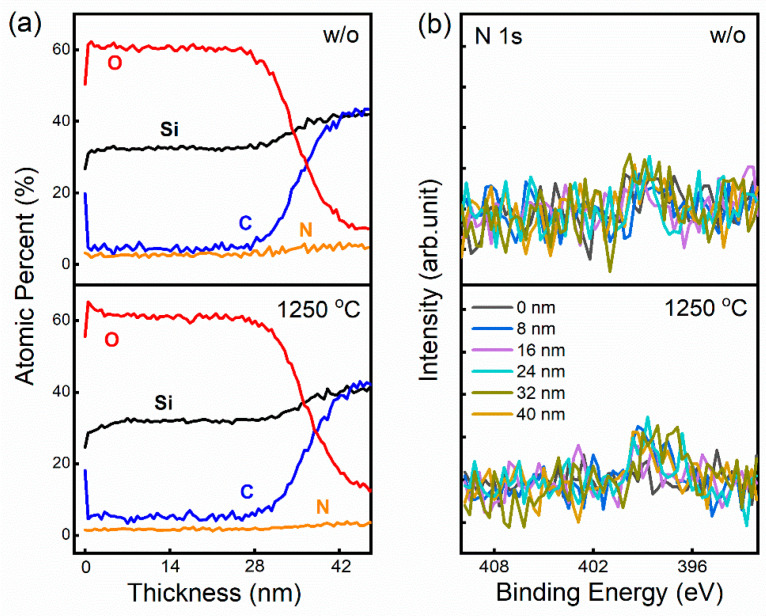
(**a**) The atomic percent of Si, O, C, and N elements and (**b**) the N 1s peak of the samples without and with annealing at 1250 °C.

In order to confirm that N_2_ only acts as a protective gas without participating in the reaction during the annealing process, XPS characterization was used to analyze the elemental composition of the SiO_2_/SiC gate stack. The in situ etching of the Ar^+^ ion beam was used to gradually remove the oxide layer. Figure 2 shows the N 1s spectra and atomic percent of Si, O, C, and N elements of the samples without and with annealing in N_2_ at 1250 °C. The XPS result reveals that no N 1s peak can be detected either in the bulk of SiO_2_ or at the SiO_2_/SiC interface for samples without and with annealing. It confirms that the annealing process does not introduce N atoms into the SiO_2_/SiC stack and N_2_ only acts as a protective gas.

## 3. Results and Discussion

### 3.1. Effect of Low Oxygen Partial Pressure Annealing at Different Temperatures

**Figure 3:**
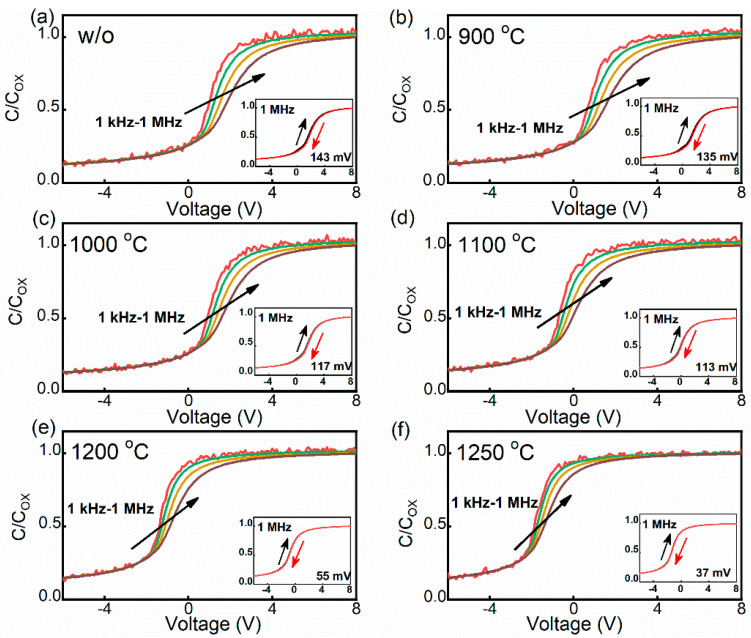
The multi-frequency C-V curves from 1 kHz to 1 MHz for the samples (**a**) without annealing and with annealing at (**b**) 900 °C, (**c**) 1000 °C, (**d**) 1100 °C, (**e**) 1200 °C, and (**f**) 1250 °C. Bidirectional C-V curves measured at 1 MHz are shown in the inset.

Figure 3a–f show the multi-frequency C-V curves of the samples without and with annealing at 900–1250 °C. The process of charging and discharging of the traps at the interface occurs during the C-V measurements, resulting in the stretching out of the C-V curves in terms of voltage [23,24], which has an effect on the dispersion of C-V curves. In addition, the charge trapping of the defects near the interface under bias stress leads to the instability of the flat band voltage [25], and the defects can be evaluated by the hysteresis at high frequency [25,26]. Therefore, the frequency dispersion and hysteresis of the C-V curves can qualitatively represent the defects at and near the interface. The large frequency dispersion and hysteresis imply the existence of a high-defect value. Comparing with the unannealed sample, the annealed samples have smaller frequency dispersion and hysteresis, which indicates that the annealing process is effective in improve the quality of the SiC MOS. Additionally, the frequency dispersion and the hysteresis decrease with increasing POA temperature, indicating that the annealing effect is temperature-dependent. In the range of 900–1250 °C, the improvement effect is more pronounced with the increase in temperature.

**Figure 4:**
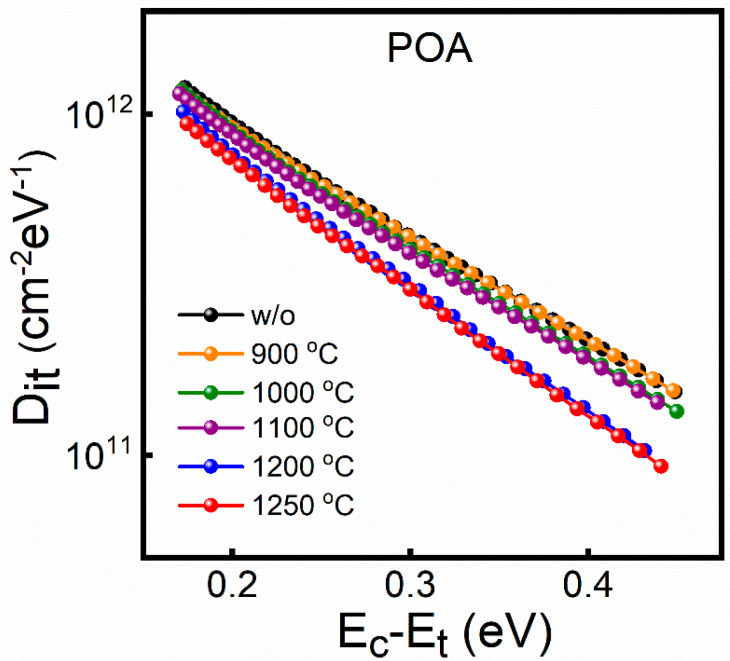
The D_it_ calculated by the high–low method for the sample annealed from 900 to 1250 °C, respectively.

In order to further quantitatively analyze the interface defects, the D_it_ value evaluated by the high–low C-V method is shown in Figure 4. Compared to the unannealed samples, the D_it_ values of the annealed samples decrease slightly with increased temperature, within the temperature range of 900–1100 °C, and decrease significantly at ≥1200 °C. The interface quality can be evaluated by the D_it_ value. The decrease in the D_it_ value indicates that the defects at the interface are repaired during the annealing process, which leads to the improvement in the interface quality. Therefore, the change trend of D_it_ proves that the annealing process with a low oxygen partial pressure improves the interface quality, and the improvement effect is more obvious at ≥1200 °C.

**Figure 5:**
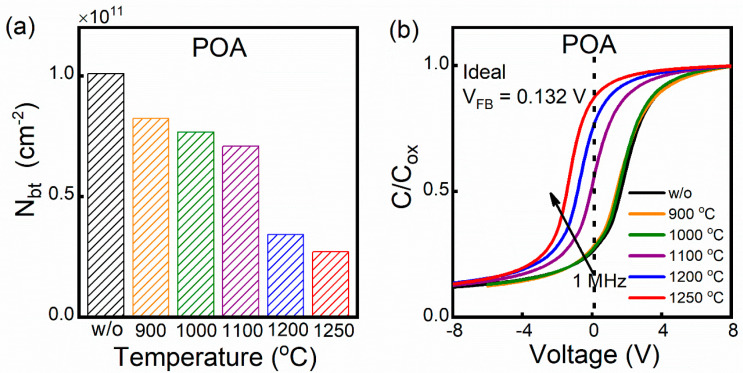
(**a**) Border trap density (N_bt_) and (**b**) 1 MHz C-V curves of annealed samples at different temperatures.

In order to assess the oxide layer quality, the defects near the interface and the shift of the V_FB_ of the samples were investigated. The defects near the interface can be quantitatively evaluated by border trap density (N_bt_), which can be estimated according to the integration area of the 1 MHz bidirectional C-V curves [27], as shown in the inset of Figure 3. In Figure 5a, the N_bt_ decreases with the increase in annealing temperature. There is a significant reduction in N_bt_ at 1200 °C, which is consistent with the trend of D_it_ in Figure 4. Therefore, for the repair of defects at and near the interface, the effect of low oxygen partial pressure annealing is significant when the temperature is up to 1200 °C. The change in the oxide layer charge can be analyzed by comparing the shift between the V_FB_ of the annealed samples and the ideal value. Figure 5b shows the 1 MHz C-V curves of the annealed samples at different temperatures and a shift in the V_FB_ from the ideal value can be observed. The 1 MHz C-V curve of the annealed samples shifts to the left with increasing temperature, suggesting that negative charge is removed during the annealing process. In other words, the low oxygen partial pressure annealing process is beneficial for the repair of charge defects in the oxide layer. It is worth noting that the charges in the oxide layer are generated in the process of thermal oxidation [28], which is related to the traps at and near the interface [29,30]. In addition, the repair of the oxide layer charge during the annealing process is not significant at <1100 °C, as indicated by the lateral shift of the C-V curve.

The above analysis shows that both the D_it_ and N_bt_ values of the annealed samples are decreased compared to the unannealed samples and the oxide layer charge also can be repaired, indicating that low oxygen partial pressure annealing can reduce traps and improve the quality of the interface and oxide layer. From the point of view of the dependence of the improvement effect and temperature, it is necessary to reach a certain temperature (≥1200 °C) for the improvement effect to be significant.

### 3.2. Annealing Mechanism of Low Oxygen Partial Pressure at Different Temperatures

**Figure 6:**
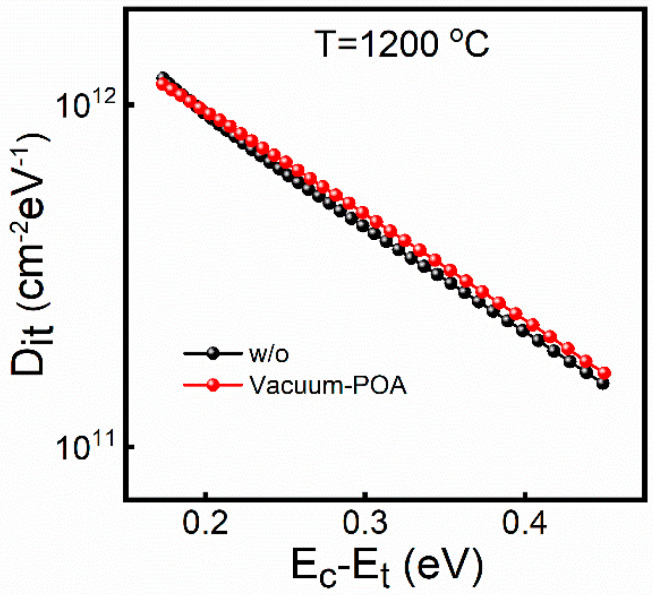
The D_it_ of the samples without and with annealing in vacuum at 1200 °C.

Based on the role of oxygen and the improvement in the interface quality, we speculate that there are two possible repair mechanisms: volatilization, and the filling of oxygen vacancies (V[O]). It has been reported that the volatilization of V[O] at the interface can reduce the D_it_ value when the samples are annealed in an anoxic atmosphere [31]. To verify whether the V[O] volatilization applies to the low oxygen partial pressure annealing mechanism, the sample annealed in the vacuum (3 × 10^−3^ Pa) at 1200 °C was used as the control group. The vacuum is conducive to the volatilization of oxygen vacancies because it contains a lower oxygen partial pressure. Therefore, if the V[O] volatilization is the dominant mechanism, the D_it_ value should be reduced after vacuum annealing at the same temperature. However, as shown in Figure 6, samples without and with vacuum annealing have almost the same D_it_, indicating that the V[O] volatilization is not the dominant mechanism during the annealing process.

**Figure 7:**
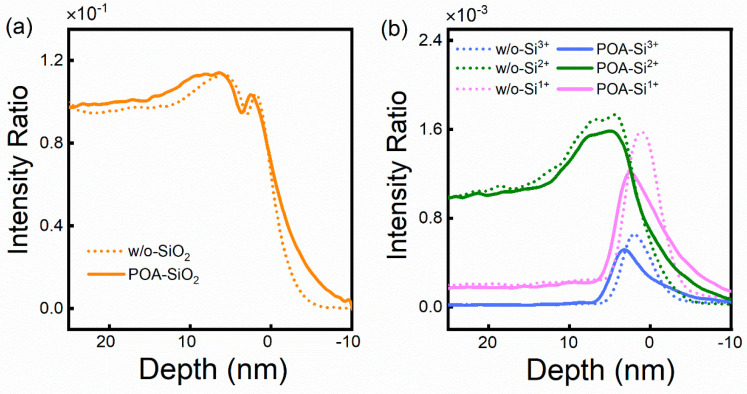
The depth distribution of oxides corresponding to the valence states (**a**) Si^4+^, (**b**) Si^3+^, Si^2+^, and Si^1+^ of Si, respectively.

In order to analyze the possibility of the V[O] filling, ToF-SIMS characterization was performed to obtain the depth distribution of silicon oxides with different chemical structures. Figure 7a shows the intensity ratio of SiO_2_ (Si^4+^) in the oxide layer of the sample without and with annealing at 1200 °C. The depth is the distance between the detection position and the SiO_2_/SiC interface, and the interface position is defined as a depth of 0 nm. Compared with the unannealed samples, the SiO_2_ ratio increases for the annealed samples, indicating that the quality of the oxide layer is improved during the annealing process. Figure 7b shows the depth distribution of oxides corresponding to the intermediate states Si^3+^, Si^2+^, and Si^1+^ of Si, respectively, which represent the V[O] distribution. The intermediate oxides of both samples have a ratio peak near the interface, indicating that the number of V[O] is the highest near the interface. The intensity ratio peak of the intermediate oxide of the annealed samples is lower than that of the unannealed samples, indicating that the V[O] can be reduced during the annealing process. Based on the above analysis, we suggest that the V[O] filling near the interface is the dominant mechanism due to low oxygen partial pressure. The V[O] near the interface is filled more by O_2_ in the annealing atmosphere with the increase in temperature and the V[O] decreases significantly at ≥1200 °C, which is consistent with the trend of the values of D_it_ and N_bt_ as a function of annealing temperature.

## 4. Conclusions

In summary, low oxygen partial pressure annealing at different temperatures has been demonstrated to be an effective way to improve the interface quality of the SiO_2_/SiC gate stack. The decrease in the D_it_ and N_bt_ values show that the defects are repaired during the annealing process. Both of the D_it_ and N_bt_ values decrease significantly, implying that the improvement effect is more obvious at ≥1200 °C. The XPS results show that no N 1s peak is detected in the oxide layer of the annealed sample, which confirms that N_2_ only acts as a protective gas and does not participate in the passivation reaction. The mechanism of V[O] volatilization is excluded due to almost the same D_it_ value for the samples without and with annealing in the vacuum. The ToF-SIMS analysis results show an increased intensity ratio of SiO_2_ in the oxide layer and a decreased intensity ratio of intermediate state oxides at the interface for the sample annealed at 1200 °C. Therefore, we infer that oxygen filling V[O] is the dominant mechanism of the interface improvement during the low oxygen partial pressure annealing process and more V[O] are filled as the temperature increases in the range of 900–1250 °C.

## Data Availability

Data are contained within the article.
